# The Value of Fruits

**Published:** 1898-03

**Authors:** 


### THE VALUE OF FRUITS.

*Modern Medicine* states regarding fruits as follows:

Fruits are, however, of great value in many forms of disease, because of the acids which they contain. These acids when taken into the blood break up some of the compounds of waste substances which have been formed, and thus give rise to an increased excretion of these substances through the kidneys. In this way fruits are a great advantage in the treatment of rheumatism, gout, gravel, and all the different morbid conditions which accompany the so-called uric-acid diathesis. The observations of Haig respecting the relation of uric acid to neurasthenia give to fruit a great dietetic value in this disease. He has shown that neurasthenia is almost always the result of the accumulation within the system of tissue wastes largely in the form of uric acid. The free use of fruits aids in the elimination of these poisons, not only by breaking up the compounds which they form within the body, but by stimulating the kidneys to increased normal activity.

Remembering the interesting fact pointed out by Bouchard, that rheumatism is really a toxemia, resulting from the decomposition of food-stuffs in a dilated or prolapsed stomach, we may also attribute the beneficial effects of a fruit diet in rheumatism and allied conditions to its value in suppressing the formation of poisonous substances in the alimentary canal in the manner already pointed out.

Obesity, which is, like rheumatism, a diathesis, may be successfully treated by a fruit dietary. This is due not only to the fact that fruit is a natural food, and thus aids the system to establish normal tissue metamorphosis and a normal balance between the processes of assimilation and disassimilation, but also because it affords a very comfortable means of reducing the amount of nutrient material received to a minimum quantity.

Fruit is chiefly water, the amount of nutrient material it contains varying from five to eight or ten per cent. in most fruits, rising to a higher figure only in dried fruits, such as dried grapes, prunes, dates, etc. The writer has succeeded in reducing excessive weight in the most satisfactory manner, by prescribing a diet consisting almost exclusively of grapes or apples, allowing only a small bit of thoroughly dried bread or zwieback in connection with the fruit. In some cases the fruit may be allowed as often as three or four times a day, if necessary to relieve an uncomfortable sensation of emptiness.

In fevers, fruits, especially in the form of fruit juices, are a most convenient and certainly the most appropriate of all foods. It is now almost universally recognized that beef tea and meat preparations of all sorts should be wholly proscribed in cases of fever, as the patient is already suffering from the accumulation of waste matters to such a degree that the addition of even the small amount contained in beef tea or a small piece of meat, may be sufficient to give rise to an exacerbation of the disease and lessen the patient's chances for recovery.

